# Mitochondrial Function and Signaling to Regulate Cellular Life

**DOI:** 10.3390/life13040975

**Published:** 2023-04-09

**Authors:** Rafael A. Casuso

**Affiliations:** Faculty of Health Sciences, Universidad Loyola Andalucía, 14004 Córdoba, Spain; rcasuso@uloyola.es

Mitochondria are essential organelles found in nearly all eukaryotic cells, responsible for producing the energy that drives cellular processes. In recent years, researchers have discovered that mitochondria are also involved in a wide range of cellular signaling pathways [1,2]. As such, unravelling the function and signaling of mitochondria is critical to understanding cellular life. This special issue, entitled “Mitochondrial Function and Signaling to Regulate Cellular Life,” brings together a collection of 7 scientific papers focused and 3 reviews on the latest research into mitochondria and their role in cellular function. The papers in this issue cover a wide range of topics, from the role of mitochondria in metabolic regulation to the impact of mitochondrial dysfunction on aging and disease.

One of the key themes that emerges from these papers is the importance of lifestyle factors in regulating mitochondrial function and signaling. For example, the close relationship between lifestyle and mitochondrial is reviewed in the paper by Vargas-Mendoza et al. [3] with a special focus on the interplay between nutrition and exercise. Another study [4] highlights the potential applications of mitochondrial biology in the context of osteoarthritis treatment. The results suggest that moderate aerobic exercise with a high-protein diet can alleviate OA symptoms and articular cartilage degradation by reducing inflammation and oxidative stress. Moreover, the paper by Casuso et al. [5] a high physiological dose of a powerful antioxidant (i.e., the polyphenol hydroxytirosol) can enhance skeletal muscle mRNA transcripts of the glucose oxidation pathway but hampers the protein translation. Thus highlighting that caution should be taken when analyzing the effects of nutritional interventions on muscle mRNA levels. In addition, Márquez-Ramírez et al. [6] shows that avocado oil can be a promising therapeutic approach at preventing hypertensive renal damage. The authors shows that a possible the possible underlying mechanism is related with decreased mitochondrial reactive oxygen species (ROS) generation and improved mitochondrial glutathione’s redox state.

Several papers in this issue explore the role of mitochondria in longevity and age-related diseases. The review by Garone et al. [7] the ATP synthase and its role in maintaining cellular function. The authors explain the role of mitochondrial transition pore on neuronal cell death in vivo and in vitro models of neurodegenerative diseases. Furthermore, another paper updates recent advances in mitochondrial quality control mechanisms that are activated in the protection conferred by different cardiac conditioning interventions [8]. It also discusses the role of extracellular vesicles in mitochondrial protection and turnover of these organelles. Concluding that modulation of mitochondrial quality control mechanisms and recognition of mitochondrial targets could provide a potential and selective therapeutic approach for intermittent ischemia/reperfusion-induced mitochondrial dysfunction. Krstic et al. [9] investigated the impact of pulmonary artery hypertension on mitochondrial function in right ventricular cardiomyocytes. For that purpose, they developed a new technique to measure beat-to-beat mitochondrial Ca^2+^ fluxes and determine mitochondrial abundance and function. They found that compensatory hypertrophy resulted in larger mitochondrial Ca^2+^ transients, indicating a compensatory mechanism to match ATP supply to the increased energy demands of hypertrophic cardiomyocytes.

In another study, Morales-García et al. [10] address the regulation of the mitochondrial unspecific pore (ScMUC) in the yeast Saccharomyces cerevisiae. Regulation of ScMUC was evaluated in isolated mitochondria under different conditions. The results showed that ScMUC opening was reversible, and it was mediated by the ATP/ADP ratio and [Ca^2+^]. A high ATP/ADP ratio promoted opening, while an increase in [Ca^2+^] closed ScMUC. Notably, closure of ScMUC in the absence of ATP synthesis resulted in an increase in ROS. These findings shed light on the regulation of mitochondrial function in yeast and provide potential targets for further research on mitochondrial function in other organisms. In another study using Saccharomyces cerevisiae the authors found that the combination of polyunsaturated fatty acids (PUFA) and ethanol hypersensitizes yeast to necrotic cell death by exacerbating membrane damage and mitochondrial cardiolipin loss, independent of mitochondrial dysfunction and ROS generation [11]. This is a study with a high biotechnological application for instance for the engineering of yeast for PUFA production and highlights the need to target both ROS production and lipid peroxidation to improve yeast resistance against necrotic cell death. Finally, a paper by Crola Da Silva et al. [12] proposes a reliable and well-characterized method for the multiparametric analysis of isolated single mitochondria by flow cytometry (FC) in the context of myocardial infarction. Using a rat model of ischemia-reperfusion and a protective approach of post-conditioning using low reperfusion pressure, they highlight FC as a reliable and sensitive method to investigate changes in mitochondrial functions and morphology in pathological conditions that disrupt their activity, such as ischemia-reperfusion.

In summary, mitochondrial research is essential for understanding physiological processes related to exercise, nutrition, and aging in both health and disease. The implementation of new techniques to study mitochondria in this context will advance our knowledge of these organelles.
